# Pathological phase transitions in ALS-FTD impair dynamic RNA–protein granules

**DOI:** 10.1261/rna.079001.121

**Published:** 2022-01

**Authors:** Natalia B. Nedelsky, J. Paul Taylor

**Affiliations:** 1 Department of Cell and Molecular Biology, St. Jude Children's Research Hospital, Memphis, Tennessee 38105, USA; 2 Howard Hughes Medical Institute, Chevy Chase, Maryland 20815, USA

**Keywords:** condensation, RNP granule, ALS-FTD

## Abstract

The genetics of human disease serves as a robust and unbiased source of insight into human biology, both revealing fundamental cellular processes and exposing the vulnerabilities associated with their dysfunction. Over the last decade, the genetics of amyotrophic lateral sclerosis (ALS) and frontotemporal dementia (FTD) have epitomized this concept, as studies of ALS-FTD-causing mutations have yielded fundamental discoveries regarding the role of biomolecular condensation in organizing cellular contents while implicating disturbances in condensate dynamics as central drivers of neurodegeneration. Here we review this genetic evidence, highlight its intersection with patient pathology, and discuss how studies in model systems have revealed a role for aberrant condensation in neuronal dysfunction and death. We detail how multiple, distinct types of disease-causing mutations promote pathological phase transitions that disturb the dynamics and function of ribonucleoprotein (RNP) granules. Dysfunction of RNP granules causes pleiotropic defects in RNA metabolism and can drive the evolution of these structures to end-stage pathological inclusions characteristic of ALS-FTD. We propose that aberrant phase transitions of these complex condensates in cells provide a parsimonious explanation for the widespread cellular abnormalities observed in ALS as well as certain histopathological features that characterize late-stage disease.

## INTRODUCTION

In eukaryotic cells, a large fraction of intracellular RNA is associated with RNA-binding proteins in ribonucleoprotein (RNP) granules, which are complex assemblies that arise via biomolecular condensation and can greatly influence the distribution and utilization of RNA (Tauber et al. 2020). These condensates vary widely in their subcellular location, composition, and size, reflecting their diverse functions in governing RNA metabolism. The functions of RNP granules are predicted to be intimately linked to their dynamism and, by extension, their material properties, which include surface tension, viscosity, and elasticity (Elbaum-Garfinkle et al. 2015). Indeed, the biophysical state of a given RNA molecule—whether it is dispersed or within a condensate, and the material properties of that condensate—likely dictate its utilization (e.g., translation) and its fate (e.g., degradation) (Adekunle and Hubstenberger 2020). Condensation of RNP granules is driven by phase transition mediated by multivalent interactions between and among RNA molecules and RNA-binding proteins (Mittag and Parker 2018).

Evidence accumulated over many years from the disciplines of genetics, cell biology, and pathology has revealed that disturbances in RNA metabolism are important contributors to both the initiation and progression of important neurodegenerative diseases, most prominently amyotrophic lateral sclerosis (ALS) and frontotemporal dementia (FTD) (Nussbacher et al. 2019). These disturbances have been observed at multiple levels of RNA metabolism, from biogenesis and splicing of nascent transcripts in the nucleus to transport and translation of mature species in the cytoplasm. Notably, experimental investigations have highlighted aberrations in phase transitions as a common mechanistic link across these wide-ranging defects (Mathieu et al. 2020). Thus, although the full pathogenic course of ALS-FTD is undoubtedly multifactorial, converging evidence suggests that a substantial fraction of ALS-FTD is caused by disturbance in properties of RNP granules and other biomolecular condensates with adverse consequences for multiple aspects of RNA metabolism, ultimately leading to neuronal dysfunction and demise. Here we review this evidence, beginning with an overview of ALS-FTD and brief discussion of relevant principles of condensate assembly and dynamics. Next, we present examples of four distinct types of disease mutations that each lead to disturbance of RNP granule dynamics and consider how these disturbances may lead to neuronal dysfunction. Finally, we conclude by speculating on the prospects for manipulating condensate dynamics to therapeutic ends.

## CLINICAL FEATURES, PATHOLOGY, AND GENETICS OF ALS-FTD

Although ALS and FTD are clinically distinct, insights from human genetics, patient pathology, and investigations into molecular mechanisms have established that the most common forms of these diseases represent two extremes of a broader disease continuum, termed ALS-FTD, that in some cases also extends to muscle tissue to cause inclusion body myopathy (IBM) (Taylor et al. 2016).

ALS, also known as Lou Gehrig's disease, is a fatal neurodegenerative disease that affects motor neurons in the brain and spinal cord. ALS can strike as early as adolescence, but is typically diagnosed in the sixth decade of life (mean age of 55 yr), when patients experience progressive deterioration of voluntary muscle function that generally culminates in death three to five years after symptom onset (Brown and Al-Chalabi 2017). In the United States, the incidence of ALS is approximately two per 100,000 people with a prevalence of approximately three to five per 100,000 people, although significant heterogeneity exists based on geography and race (Xu et al. 2020). At the cellular level, the clinical syndrome of ALS arises from impaired motor neuron function that is most attributable to collapse of axonal function. This axonopathy ultimately reflects multicellular pathology, including cell-autonomous defects in neurons aggravated by secondary defects in glial support cells such as oligodendrocytes and astrocytes, and toxicity imposed by microglial-mediated inflammation (for review, see Taylor et al. 2016). Substantial evidence also suggests that this neuronal dysfunction occurs over a protracted period of time—perhaps even decades—that precedes the ultimate demise of individual motor neurons (Moloney et al. 2014).

FTD represents a clinically and pathologically heterogeneous group of dementias characterized by progressive atrophy of the frontal and/or temporal lobes, manifesting in changes in behavior and personality or deficits in language functions (Bang et al. 2015). As defined by neuropathology, FTD has two major histopathological forms. The first, found in approximately 50% of patients with FTD, is characterized by intracellular protein deposits that stain negative for tau but positive for the RNA-binding protein TDP-43. This form of FTD has significant clinical, genetic, and pathological overlap with ALS and represents one end of the ALS-FTD continuum. The remaining ∼50% of patients with FTD have pathology marked by tau-positive, TDP-43-negative inclusions; this form of FTD comprises the “tauopathies” (e.g., Alzheimer disease, multisystem atrophy, corticobasal degeneration) and appears to be etiologically distinct from ALS.

A rare, related syndrome is multisystem proteinopathy (MSP), a dominantly inherited, pleiotropic, degenerative disorder that can affect muscle, bone, and/or the central nervous system. MSP can manifest clinically as ALS, FTD, IBM, Paget's disease of bone (PDB), or as multisystem disease (Taylor 2015). There is substantial genetic and histopathological overlap between ALS-FTD and MSP, with the latter caused by mutations in VCP, hnRNPA1, hnRNPA2B1, TIA1, matrin 3, p62, and UBLQN2 (Taylor 2015). Whereas we focus primarily on ALS-FTD below, overlapping genetics and histopathology suggest that emerging disease insights may be relevant more broadly to MSP and some forms of IBM and PDB as well (Ralston and Taylor 2019).

The presence of fibrillar TDP-43 in neuronal inclusions is a hallmark of nearly all forms of ALS-FTD (Neumann et al. 2006), with the exception of disease caused by mutations in *SOD1* or *FUS*, which result in inclusions that are TDP-43-negative but positive for SOD1 or FUS, respectively (Rosen et al. 1993; Kwiatkowski et al. 2009; Vance et al. 2009). Additional RNA-binding proteins are less frequently found deposited in pathology, including the FET proteins FUS, TAF15, and EWS (Hofmann et al. 2019) and hnRNPA3 (Fifita et al. 2017), as well as abnormal redistribution of these and additional RNA-binding proteins such as hnRNPA1 and hnRNPA2B1 from the nucleus to the cytoplasm (Thibault et al. 2021). Indeed, abnormal redistribution of RNA-binding proteins from the nucleus to the cytoplasm, including TDP-43, may be the most prominent histopathological feature of ALS-FTD (Honda et al. 2015; Koyama et al. 2016; Fifita et al. 2017) and genetically related myopathies (Picchiarelli and Dupuis 2020).

ALS-FTD caused by repeat expansions in *C9ORF72*, the most common cause of ALS-FTD, features the canonical pathology of deposition of RNA-binding proteins, including TDP-43, but has additional features that are unique to this mutation. Specifically, *C9ORF72*-related ALS-FTD neuropathology is characterized by the formation of nuclear RNA foci containing the expanded *C9ORF72* transcript (DeJesus-Hernandez et al. 2011; Renton et al. 2011) as well as prominent deposition of neomorphic polydipeptide repeats (poly-PR, poly-GR, poly-PA, poly-GP, and poly-GA) that arise from unconventional repeat-associated non-AUG (RAN) translation of the expanded *C9ORF72* transcript in multiple reading frames. Of these polydipeptides, the arginine-rich poly-PR and poly-GR (the “R-polydipeptides”) show early deposition (Saberi et al. 2017; Sakae et al. 2018), exquisite toxicity in animal models of disease (Mizielinska et al. 2014; Freibaum et al. 2015; Choi et al. 2019; Cook et al. 2020), and the closest correlation with regions of brain degeneration in patients (Vatsavayai et al. 2016).

In approximately 90% of cases, patients present with ALS-FTD without a family history. This form of disease is termed sporadic ALS-FTD, defined in contrast to familial ALS-FTD, although genetic variants are being increasingly identified in sporadic ALS-FTD in the same genes as those mutated in familial forms. Indeed, advances in genetic sequencing technology over the last 10 years have enabled rapid expansion of the list of ALS-FTD-associated genes from a single gene (*SOD1*, identified in 1993; Rosen et al. 1993) to at least 20 genes at present. The identification of these additional genetic forms of ALS-FTD provides strong evidence implicating RNA metabolism as a common contributor to disease. At least eight ALS-FTD- or IBM-associated genes encode RNA-binding proteins (TDP-43 [Kabashi et al. 2008; Sreedharan et al. 2008], FUS [Kwiatkowski et al. 2009; Vance et al. 2009], hnRNPA1 [Kim et al. 2013; Liu et al. 2016], hnRNPA2B1 [Kim et al. 2013], matrin 3 [Johnson et al. 2014], TIA1 [Mackenzie et al. 2017], hnRNPDL [Vieira et al. 2014], and annexin A11 [Smith et al. 2017; Leoni et al. 2021]); in addition, the disease-associated gene *ANG* encodes angiogenin, a tRNA processing protein (Greenway et al. 2006). An additional four disease genes modulate stress granule dynamics, disassembly, or degradation: *VCP* (Johnson et al. 2010), *OPTN* (Maruyama et al. 2010), *UBQLN2* (Deng et al. 2011), and *SQSTM1* (Fecto et al. 2011; Buchan et al. 2013; Alexander et al. 2018; Chitiprolu et al. 2018; Gwon et al. 2021; Kakihana et al. 2021). Furthermore, although no normal function has been definitively ascribed to *C9ORF72*, ALS-FTD-associated mutations in this gene lead directly to disruption of multiple RNP condensates, as detailed below.

Additional genes cluster into other functional categories, most notably axonal transport and cytoskeletal proteins; this group includes *DCTN1*, which encodes dynactin 1 (Puls et al. 2003); *PFN1*, which encodes profilin 1 (Wu et al. 2012); *KIF5A*, which encodes kinesin 5A (Nicolas et al. 2018); *TUBA4A*, which encodes tubulin β-4A (Smith et al. 2014); and tau (Hutton et al. 1998). A final group of ALS-FTD-related genes have no known relation to RNA metabolism: *SOD1* (Rosen et al. 1993), *GRN*, which encodes progranulin (Baker et al. 2006; Cruts et al. 2006), and TBK1 (Freischmidt et al. 2015).

Follow-up of these genetic insights in model systems has revealed a broad array of cellular defects. Among these, a common theme has emerged highlighting defects in RNA metabolism at multiple levels, with the most prominent and consistent evidence pointing to deficiencies in post-transcriptional processing related to a loss of function (replicated by gene knockouts) and/or defects in translation attributable to a toxic gain of function (replicated by exogenous addition of disease gene product). With regard to the former, several studies of TDP-43 have demonstrated evidence of loss of function in the nucleus related to its splicing activity, particularly in splicing of cryptic exons (Arnold et al. 2013; Ling et al. 2015), with downstream consequences for axonal function (Klim et al. 2019; Melamed et al. 2019). With regard to defects in translation, expression of disease-associated proteins has been demonstrated to impose toxic gain of function effects, including deficiencies in translation in distal compartments of neurons, as will be detailed below. Many of these and other findings converge around disturbances in the material properties and dynamics of structures that arise via biomolecular condensation, and RNP granules in particular. In the following section, we briefly review the functions and basic biophysical underpinnings of RNP granule assembly and dynamics in order to provide a framework upon which to build an understanding of disease etiology.

## KEY CONCEPTS IN BIOMOLECULAR CONDENSATION

Biomolecular condensates constitute a diverse group of intracellular structures that govern a variety of cellular functions, spanning a broad range of complexities and sizes (Banani et al. 2017). RNP granules are condensates composed of RNA and protein organized into assemblies with diameters that can span in scale from nanometers to several microns. The specific RNA and protein composition of RNP granules is distinct across their various subtypes, which include nucleoli, RNA transport granules, Cajal bodies, nuclear speckles, paraspeckles, PML bodies, germ granules, P bodies, and stress granules (Shin and Brangwynne 2017). Some RNP granules are constitutive, whereas others assemble and disassemble conditionally, such as during specific stages of development or in response to changes in cellular state. For instance, stress granules, a type of RNP granule frequently implicated by studies of ALS-FTD, are normally induced transiently in response to a variety of cellular stressors. Of note, the composition of stress granules overlaps substantially with that of RNA transport granules that arise spontaneously in some cell types, such as the distal compartments of neurons (Buchan and Parker 2009; Fallini et al. 2012; Sahoo et al. 2018; Liao et al. 2019).

Found throughout the cell, including the nucleus, cytoplasm, and distant processes of neurons, RNP granules are implicated in governing many aspects of RNA metabolism from biogenesis to splicing, localization, translation, and eventual degradation. The mechanisms proposed to underlie governance of these activities largely fall into one of two categories: either (i) sequestering constituents to limit their activity in the nucleoplasm or cytoplasm (Shin and Brangwynne 2017), which may represent a form of buffering, or (ii) creation of unique chemical environments, reaction compartments, or interfaces that promote aspects of RNA metabolism (e.g., splicing at the interface between nuclear speckles and the nucleoplasm; Liao and Regev 2021).

A role for RNP granules in RNA metabolism has been defined more extensively for some RNP granules than for others. For instance, the nucleolus is well known as the site of ribosome biogenesis—a function whose biophysical connection to phase separation has now been defined (Riback et al. 2020)—and the function of RNA transport granules in transporting mRNPs to distal sites within neurons is necessary to support local protein synthesis (for review, see Dalla Costa et al. 2021). Furthermore, Cajal bodies contribute to chromosome topology by organizing sn/snoRNA gene loci from across the genome into highly transcriptionally active intra- and inter-chromosomal regions; accordingly, disruption of Cajal bodies perturbs the expression of snRNA genes and impairs RNA maturation processes and splicing fidelity (Wang et al. 2016).

Defining the precise functions supported by other RNP granules has been more challenging. Nuclear speckles, for example, have been implicated in mRNA splicing, transcription termination, and nuclear export of mRNA, but these RNP granules have proven difficult to manipulate experimentally, as not a single factor has been identified without which speckles do not form (Ilik and Aktas 2021). Nonetheless, the subcompartmentalization of nuclear speckles implicates these structures as a site of RNA splicing. Specifically, spliceosomes are localized to the interface of these condensates with the nucleoplasm (Liao and Regev 2021). Moreover, there is a striking, nonrandom distribution of transcripts that pass into nuclear speckles: whereas introns remain at the speckle periphery with spliceosomes, spliced RNA products migrate into the speckle interior (Johnson et al. 2000; Hall et al. 2006). A compelling model to explain the role of nuclear speckles in splicing posits that exons are sequestered to the condensate interior through interaction with SR proteins, while introns are excluded from the speckles through interaction with hnRNPs. This arrangement would align splice junctions along the speckle interface, thus promoting splice site selection by spliceosomes enriched in this location (Liao and Regev 2021). Nuclear speckles have also been implicated in transcription, based on the observation that the physical proximity of heat-shock responsive genes to nuclear speckles correlates with their transcriptional output (Zhang et al. 2020). Paraspeckles, which are protein-rich nuclear assemblies built around a specific lncRNA scaffold, also appear to actively participate in the post-transcriptional processing of primary miRNAs (pri-miRNAs) (Jiang et al. 2017). PML bodies have been implicated in a wide variety of biological processes ranging from senescence to viral infections and stemness, although no unifying function has been firmly defined (Lallemand-Breitenbach and de The 2018).

However, RNP granule “function” need not be limited to circumstances in which a granule actively promotes the catalysis of a biochemical reaction. Rather, in many cases RNP granules appear to serve as a temporary storage site for specific mRNAs and RNA-binding proteins, with significant functional consequences. For example, germ granules repress mRNA expression by sequestration of certain maternal transcripts in association with repressors (Aoki et al. 2021) and also serve a protective role by shielding transcripts from small RNA-based regulatory pathways (Dodson and Kennedy 2019; Ouyang et al. 2019) and enriching mRNAs for robust germline development (Lee et al. 2020; Parker et al. 2020). With regard to P bodies, some investigations have suggested that these RNP granules represent the cellular sites of mRNA decay (Sheth and Parker 2003), whereas other studies have demonstrated that these assemblies represent storage sites for translationally repressed mRNAs and inactive mRNA decay enzymes (Stalder and Muhlemann 2009; Luo et al. 2018). The regulation of gene expression by paraspeckles has also been linked to sequestration of both RNA and protein (Chen and Carmichael 2009; Hirose et al. 2014; Imamura et al. 2014), and the nucleolus has a well-documented role acting as a protein quality control compartment by temporarily storing metastable nuclear proteins that misfold upon heat stress (Frottin et al. 2019) and is required for recovery of epigenetic regulators and maintenance of the epigenome after heat shock (Azkanaz et al. 2019).

Stress granules and RNA transport granules share similarities with germ granules in that their RNA content is enriched in nontranslating mRNAs, and this may have functional significance. Indeed, RNA transport granules not only control the spatiotemporal distribution of mRNAs, but also retain these mRNAs in a condensed state where they are inactive but poised for bursts of local protein synthesis in response to stimuli, such as activity-dependent local translation in synaptic compartments (Kosik and Krichevsky 2002; Sahoo et al. 2018). Stress granules arise as a consequence of inhibited translation rather than a mechanism of limiting translation, but have been suggested to represent a protective response to house translationally stalled mRNAs, ribosomal components, and both RNA-binding and non-RNA-binding proteins (Jain et al. 2016). Whereas the importance of this proposed protective response remains ill-defined, recently published evidence suggests that stress granule assembly mitigates the risk of irreversible RNA tangles that may accrue when cytoplasmic RNA concentrations rise abruptly, such as occurs with polysome disassembly (Guillen-Boixet et al. 2020). However, it is likely that multiple stress-responsive functions have evolved to exploit the assembly of stress granules—including service as a platform that regulates intracellular signaling cascades. For example, stress granules sequester PKC to protect against MAPK signaling hyperactivation (Kanda et al. 2021) and sequester TORC1 to regulate TORC1 signaling (Takahara and Maeda 2012). Independent of stress responsiveness, compelling evidence suggests that G3BP1-seeded RNP granules that assemble spontaneously in distal neuronal compartments negatively regulate local translation by sequestration of mRNAs and translation machinery (Sahoo et al. 2018).

Indeed, the concept of functional sequestration of specific molecules into RNP granules is intimately linked to the concept of buffering, in which the bioavailability of a given constituent is limited by sequestering excess molecules beyond a specific concentration threshold into the dense phase (Chattaraj et al. 2021). In this manner, the noise (e.g., fluctuations in concentration) in the light phase is reduced or buffered. We revisit this concept below in our discussion of how disturbances in condensate dynamics are linked to cellular dysfunction.

As reviewed elsewhere in this issue, RNP granules arise by liquid–liquid phase separation (LLPS), a process wherein certain components of a mixed phase de-mix to produce a second, more concentrated liquid phase that remains in dynamic equilibrium with the less concentrated liquid phase. Early insights into this process were derived from the serendipitous discovery in 2012 that intrinsically disordered low-complexity domains (LCDs) in many RNA-binding proteins can self-assemble into fibrils characterized by cross-β sheet content and the suggestion that this higher-order assembly contributes to the formation of RNP granules (Kato et al. 2012). Subsequent studies extended these findings by demonstrating that the LCDs in many RNA-binding proteins—including the disease-associated proteins hnRNPA1, TDP-43, and FUS (Lin et al. 2015; Molliex et al. 2015; Murakami et al. 2015; Patel et al. 2015)—can mediate LLPS. This discovery led to the appreciation that in addition to the ability of some LCDs to assemble into relatively stable structures driven by cross-β sheet formation, the sequence and patterning of many intrinsically disordered regions (IDRs) in RNA-binding proteins encode multivalent, short-duration interactions that collectively drive LLPS. Beyond revealing insights into RNP granule assembly, the discovery that IDRs frequently encode weak, multivalent interactions that can support LLPS greatly animated the field, since IDR segments are present in more than one-third of the proteome (Peng et al. 2014), suggesting that LLPS might be an organizational strategy governing broad aspects of cell biology.

Although the condensation and liquid behavior of droplets of individual RNA-binding proteins are highly reminiscent of the behavior of RNP granules where these proteins reside, simple reconstituted systems have limited ability to reveal the mechanisms of assembly of complex RNP granules in living cells, in which hundreds of constituents give rise to a bewildering number of interactions that collectively drive assembly. Indeed, whereas condensates are enriched in proteins that undergo monocomponent LLPS, the phase separation of behavior of individual proteins in vitro gives little information about the role of a given constituent in condensate assembly, material properties, or function in living cells. In one particularly germane example, ALS-causing mutations in hnRNPA1 have no impact on its phase separation behavior in vitro, but lead to dynamical arrest of RNP granules in living cells (Molliex et al. 2015). Similarly, for some mutations in TDP-43 the impact on mono-component LLPS does not correlate with functional consequences in living cells where TDP-43 undergoes condensation with RNA and other proteins (Schmidt et al. 2019; Hallegger et al. 2021).

**FIGURE 1.**
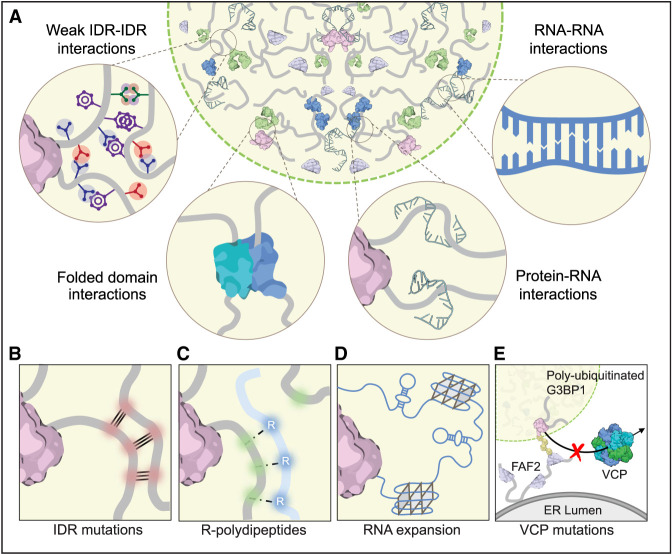
(*A*) The forces underlying condensation of RNP granules include weak IDR–IDR interactions, folded domain interactions, protein–RNA interactions, and RNA–RNA interactions. Multiple, distinct types of genetic mutations intersect upon the process of RNP granule condensation and function, giving rise to a common set of neurodegenerative diseases. Each of these types of genetic mutation increases the driving forces underlying condensation. These include (*B*) mutations in IDRs, (*C*) R-polydipeptides that bind IDRs, and (*D*) aberrant RNP condensates driven by pathological RNA expansions in *C9ORF72*-related ALS/FTD. Furthermore, (*E*) mutations in VCP lead to failed higher-order regulation of stress granule clearance.

The assembly of complex condensates such as RNP granules in living cells involves not only transient IDR–IDR interactions of RNA-binding proteins, but other modes of constituent interaction as well, including RNA–protein interactions, RNA–RNA interactions, and stable protein–protein interactions (Fig. 1A; Mittag and Parker 2018; Guillen-Boixet et al. 2020; Sanders et al. 2020; Yang et al. 2020). Great strides have been made in understanding how these interactions orchestrate the condensation that underlies RNP granule assembly. One approach to making sense of this highly complex assembly of interacting molecules of different types derives from graph theory and its concept of a *percolation threshold*, which is the threshold achieved when the sum and duration of all interactions are sufficient to create a system-spanning network (Eberle et al. 2012). Accordingly, a system of molecules that is populated by few and fleeting interactions will remain a single mixed phase. As this system is populated with additional or stronger interactions, there is a point—the percolation threshold—at which a system-spanning network is achieved, resulting in phase separation to create a low viscosity liquid. If the system is populated with additional or stronger interactions, these interactions change the material properties of the dense phase to create a higher viscosity liquid, and may even reach a second threshold to produce a liquid-to-solid phase transition (e.g., fibril formation).

Applying this concept to RNP granules, the creation of a system-spanning network is represented by the collective interactions of proteins and RNAs that result in LLPS, creating an RNP granule as a distinct subcellular compartment. In this context, the factors that determine the system state are (i) the concentrations of the individual constituent molecules, (ii) the number of interactions per molecule, and (iii) the lifetime of those interactions (Mathieu et al. 2020). This perspective gives rise to the *network-encoded percolation model* of RNP granule assembly, wherein the relative contribution of individual interactions toward reaching the percolation threshold is encoded within the topology of each granule's distinct network of interactions (Mathieu et al. 2020; Yang et al. 2020). By logical extension, the contribution of any individual molecule to phase separation should reflect its centrality within the constituent network.

This model is consistent with empirical observations that each type of RNP granule represents a unique biomolecular network with specific topology. Moreover, the forces underlying granule assembly are distributed unevenly across this network of interacting molecules—that is, some nodes within a given network have greater influence on granule assembly than others. For example, our recent study of stress granules defined a core protein–RNA network and correlation between the network centrality of a given node and its contribution to assembly (Yang et al. 2020). Importantly, the most central nodes also play an outsized role in establishing the composition, and therefore the identity, of distinct subtypes of RNP granule. For example, the most central node in the stress granule network is the RNA-binding protein G3BP1 (Yang et al. 2020), whereas the most central node in the granular component of nucleoli is NPM1 (Mitrea et al. 2016). Accordingly, titrating increasing concentrations of G3BP1 into a complex whole-cell lysate eventually results in LLPS to form a complex condensate that faithfully recapitulates the protein and RNA composition of stress granules, as assessed by mass spectrometry proteomics and RNA sequencing. In contrast, titrating increasing concentrations of NPM1 into a complex whole-cell lysate culminates in LLPS to form a complex condensate that faithfully recapitulates the protein and RNA composition of nucleoli (Freibaum et al. 2021). Thus, highly central nodes play a key role in establishing a specific network topology and subsequent condensate identity. These concepts have direct applicability to understanding how disease mutations affect condensate assembly and dynamics, as well as prospects for manipulating condensate behavior experimentally or therapeutically.

## CONVERGING EVIDENCE OF ALTERED RNP GRANULE DYNAMICS IN DISEASE

Our current understanding of the etiology and pathogenesis of ALS-FTD derives from decades of investigation of the underlying genetics and histopathology of this spectrum of diseases, supplemented by investigations in model systems. The view arising from these observations is that the dysfunction and ultimate demise of motor axons reflects their status as a single point of failure within a complex physiological motor system that comprises multiple cell types, including both neurons and glia. Whereas cell-autonomous defects in motor neurons are certainly central to disease, abnormalities in other cell types are important and perhaps essential contributors, including interneurons, astrocytes, oligodendrocytes, microglial cells, and perhaps muscle cells. Thus, “motor neuron disease” represents the culmination of multiple types of insults impacting multiple types of cells.

Although a plethora of defects in biological processes have been recorded in disease models, the most consistently observed and most proximal to the disease-initiating mutations are disturbances of RNA metabolism, including post-transcriptional processing, RNA export from the nucleus, trafficking of RNAs, biogenesis of noncoding RNAs, the synthesis of ribosomal subunits, translation, and degradation of RNAs (Nedelsky and Taylor 2019). Among these, perhaps the most consistently observed are defects in nuclear RNA splicing and cytoplasmic RNA translation. Considering the wide variety of defects in RNA metabolism, the appreciation that much of this biology is governed by condensation, and the discovery that ALS-FTD-causing mutations disturb the dynamism of RNP condensates, the most parsimonious view is that defects in condensation underlie many of the defects in RNA metabolism that are characteristic of these diseases.

The recognition that abnormal condensation contributes to ALS-FTD arises from the appreciation that many ALS-FTD-causing mutations fall into two general classes. In the first, mutations directly impact constituents of RNP granules, changing their interactions in a manner that alters the assembly and material properties of the condensates in which they reside. In the second, mutations occur in factors that exert higher-order regulation over these same constituents, with the same eventual consequences to granule dynamics. Below we review three examples of the first class—mutations in the LCDs of RNA-binding proteins, neomorphic peptides that exploit common modes of interaction underlying condensation, and pathological amplification of RNA–RNA interactions through expansion of GC-rich RNA sequences (Fig. 1B–D)—followed by one example of the second class, represented by mutations in VCP (Fig. 1E).

### LCD mutations in RNA-binding proteins

As noted above, some of the earliest insights into the phase separation behavior of biomolecules highlighted a central role for the LCDs of RNA-binding proteins in mediating fibril assembly in vitro (Kato et al. 2012). Immediately following this report, ALS-FTD-causing mutations were identified in the LCDs of hnRNPA1 and hnRNPA2B1 that reduced the concentration necessary for this phase transition and accelerated the formation of fibrils (Kim et al. 2013). Indeed, disease-causing mutations in many RNA-binding proteins conspicuously cluster in LCDs in association with ALS, FTD, IBM, or a combination of these conditions, a pattern that has been observed for not only hnRNPA1 and hnRNPA2 (Kim et al. 2013, 2021; Liu et al. 2016; Qi et al. 2017; Naruse et al. 2018; Beijer et al. 2021) but also TDP-43 (Sreedharan et al. 2008), FUS (Kwiatkowski et al. 2009), TIA1 (Klar et al. 2013; Mackenzie et al. 2017), matrin 3 (Johnson et al. 2014), and hnRNPDL (Vieira et al. 2014).

The manner in which LCD mutations disturb RNP granule assembly and dynamics is strikingly similar across different disease genes. For hnRNPA1 and hnRNPA2B1, LCD mutations accelerate the formation of labile fibrils in vitro and alter the dynamics of stress granule assembly in cells (Kim et al. 2013), an observation placed into context with the recognition that the LCD of hnRNPA1 contributes to stress granule assembly and that disease mutations pathologically enhance this process (Molliex et al. 2015). Other studies of hnRPNA1 (Lin et al. 2015), TDP-43 (Conicella et al. 2016), TIA1 (Mackenzie et al. 2017), and hnRNPDL (Batlle et al. 2020) have echoed this finding, consistently showing that disease mutations exploit the LCD-based interactions that regulate the assembly and material properties of stress granules, causing a leftward shift in percolation thresholds for both LLPS and liquid-to-solid phase transition. These lowered thresholds result in a greater propensity for condensation, the formation of more viscous condensates with reduced dynamics, and excess liquid-to-solid phase transitions that can produce the fibrillar pathology observed in patient cells (Fig. 1B).

**FIGURE 2.**
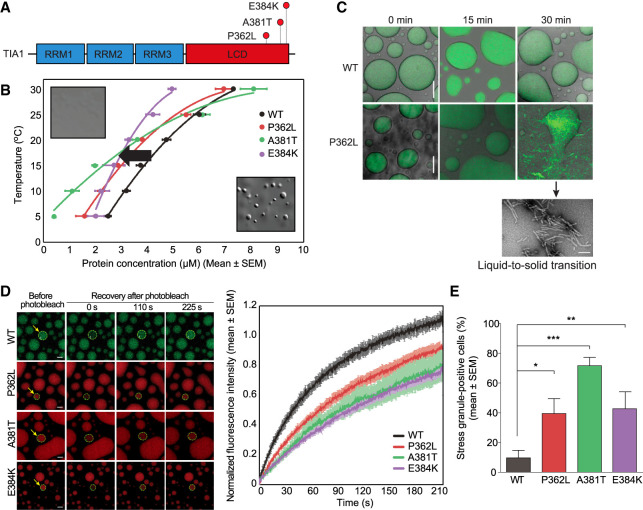
The introduction of LCD mutations in TIA1 consistently reduces the percolation threshold for phase separation in vitro by strengthening homotypic LCD interactions. (*A*) Three disease-causing mutations are shown in the LCD of TIA1: P362L and A381T, which are associated with ALS, and E384K, which causes Welander distal myopathy. (*B*) Phase diagrams showing coexistence lines for indicated purified TIA1 proteins. *Inset* images show representative DIC images of single-phase (*upper left*) and two-phase (*lower right*) solutions of WT TIA1. Disease-associated LCD mutations caused a leftward shift in the coexistence line (black arrow) to a lower protein concentration, indicating a heightened propensity for mutant TIA1 to undergo LLPS. (*C*,*D*) Predictably, the dynamism of TIA1 droplets in vitro is also significantly reduced (*D*, FRAP showing reduced mobility of mutant TIA1 in the dense phase), with the percolation thresholds shifted low enough that some liquid-to-solid phase transitions occur, as reflected by the formation of amyloid fibrils (*C*). (*E*) As TIA1 is a major constituent and promoter of stress granule assembly, these strengthened interactions reverberate throughout the stress granule network and change its dynamics, resulting in accumulation of persistent, nondynamic, TDP-43-containing stress granules (data reproduced with permission from Mackenzie et al. 2017).

Among these, TIA1 provides a striking example of how a single point mutation in a single constituent of stress granules is sufficient to slow the dynamics of disassembly and cause the accumulation of nondynamic granules (Fig. 2; Mackenzie et al. 2017). Notably, TIA1 is also a constituent of different types of condensates, including stress granules and RNA transport granules, although it is a predominantly nuclear protein and may also reside in some nuclear condensates. Thus, the consequences of TIA1 mutations likely impact multiple types of condensates, although it remains to be determined the relative contribution of altered dynamics of these different condensates to the cellular dysfunction that culminates in ALS.

Studies of the RNA-binding proteins TDP-43 and FUS have effectively tracked the consequences of ALS-FTD-causing LCD mutations from their most fundamental, direct effects on LLPS of purified recombinant protein to their complex neurotoxic effects within a physiological system. Specifically, mutations in TDP-43 that reduce the concentration threshold for LLPS (Conicella et al. 2016) and increase the propensity for liquid-to-solid phase transitions (Johnson et al. 2009) impair the dynamics and trafficking of TDP-43-laden granules in neuronal axons (Alami et al. 2014). Similarly, LCD mutations in FUS reduce the concentration threshold for LLPS, accelerate liquid-to-solid phase transition in vitro (Patel et al. 2015), and result in irreversible FUS assemblies that sequester RNP granule cargo in cells (Murakami et al. 2015). Bridging the gap to in vivo systems, subsequent experiments showed that sequestration of these RNP granule components results in decreased local protein synthesis by RNP granules in neuronal axon terminals as well as accompanying deposits of FUS within *Caenorhabditis elegans* neurons (Murakami et al. 2015).

### Mutations in *C9ORF72*

Pathological microsatellite expansions of a GC-rich motif in the first intron of the *C9ORF72* gene are the most common cause of ALS-FTD, accounting for up to 50% of familial cases and up to 10% of sporadic cases. Studies of mutations in *C9ORF72* have revealed additional, unexpected routes by which disease-causing mutations perturb biomolecular condensation, resulting in the same clinical phenotype as diseases initiated by LCD mutations. Pathological expansion in *C9ORF72* results in two toxic intermediates that mediate disease. The first are abnormal condensates that arise in the nucleus triggered by the retained intronic RNA sequences harboring an elongated G_4_C_2_ repeat. The second are neomorphic polydipeptide repeats that arise via RAN translation when transcripts with these retained introns reach the cytoplasm. The expanded GC-rich repeat is bidirectionally transcribed and both the sense and antisense GC-rich transcripts undergo translation in multiple frames (Ash et al. 2013; Mori et al. 2013; Zu et al. 2013); thus, C*9ORF72* mutations give rise to six distinct polydipeptide products. In patients, degenerating brain regions are heavily laden with these pathological RNA foci and polydipeptides (DeJesus-Hernandez et al. 2017; Saberi et al. 2017; Sakae et al. 2018). As mentioned above, empirical studies in model systems and correlative studies in human pathology have strongly implicated the R-polydipeptides (poly-GR and poly-PR) as drivers of disease.

Remarkably, unbiased global proteomics revealed a striking propensity of poly-GR and poly-PR to bind directly to LCDs in many RNA-binding proteins, including all those known to harbor LCD mutations that are causative of ALS-FTD (Lee et al. 2016), likely reflecting the high enrichment in these LCDs of residues capable of interacting with arginine via π–cation, charge–charge, and even π–π interactions (Vernon et al. 2018; Das et al. 2020). Moreover, the presence of R-polydipeptides was found to significantly reduce the LLPS concentration threshold and alter the material properties of simple condensates formed from these RNA-binding proteins (Haeusler et al. 2014; Kwon et al. 2014; Lee et al. 2016; Boeynaems et al. 2017; White et al. 2019), implicating disturbance of condensate properties in cells. Indeed, these R-polydipeptides were found to insinuate themselves into multiple RNP condensates, strengthening interactions between constituents of the condensates and disturbing their material properties in a manner precisely analogous to LCD mutations in RNA-binding proteins (Fig. 1C; Haeusler et al. 2014; Kwon et al. 2014; Lee et al. 2016; Boeynaems et al. 2017; White et al. 2019). Accordingly, R-polydipeptides cause widespread disturbances in the dynamics of multiple condensates, including the central channel of the nuclear pore, stress granules, RNA transport granules, and nucleoli (Haeusler et al. 2014; Kwon et al. 2014; Wen et al. 2014; Lee et al. 2016; Boeynaems et al. 2017; Shi et al. 2017; Zhang et al. 2018), which likely underlie observed disturbances in nucleocytoplasmic transport (Freibaum et al. 2015; Jovičić et al. 2015; Zhang et al. 2015), altered RNA splicing (Prudencio et al. 2015), impaired trafficking of RNA transport granules (Burguete et al. 2015), and impaired translation (Kanekura et al. 2016). In the case of nucleoli, R-polydipeptides directly interact with the acidic domain of NPM1, thereby displacing the native arginine-rich partners of NPM1 and altering the dynamism of the granular component of the nucleolus (Lee et al. 2016). The functional consequence is impaired flow of rRNA through the granular component with massive engorgement of rRNA in the dense fibrillar component of the nucleolus (Lee et al. 2016). Thus, the altered material property of the nucleolus results in functional impairment: namely, reduced ribosome biogenesis and maturation (Haeusler et al. 2014; Kwon et al. 2014; Lee et al. 2016; White et al. 2019). R-polydipeptides also disrupt the normal role of NPM1 in protein quality control within the nucleolus (Frottin et al. 2019) and in double-stranded break repair of DNA (Andrade et al. 2020), although the precise mechanisms remain to be established.

### RNA expansions

An additional point of convergence between RNP granules and neurodegenerative disease was revealed by the appreciation that disease-associated RNA can undergo LLPS even in the absence of protein (Jain and Vale 2017) and that these RNA–RNA interactions contribute to RNP granule assembly (Van Treeck and Parker 2018; Van Treeck et al. 2018). Indeed, disease-associated RNA expansions, a frequent cause of inherited neurodegeneration, can promote pathological RNA-driven condensation where the length threshold for phase transition in vitro matches the length threshold at which RNA foci form in cells, which also tracks with the length at which disease symptoms manifest in patients (Fig. 1D; Jain and Vale 2017). These observations may have direct relevance to a range of neurodegenerative diseases characterized by the presence of RNA foci, including myotonic dystrophy types 1 and 2, Huntington disease, Huntington disease-like 2, fragile X-associated tremor/ataxia syndrome, spinocerebellar ataxia types 3, 8, 10, 31, and 36, and Fuchs endothelial corneal dystrophy (for review, see Zhang and Ashizawa 2017).

### Mutations in VCP

As noted above, mutations in VCP are associated with MSP, a disease spectrum that ranges from ALS to FTD and IBM (Watts et al. 2004; Johnson et al. 2010; Taylor 2015). These mutations appeared for some time to be only peripherally related to RNA metabolism, as the known function of VCP—a molecular chaperone that segregates ubiquitinated substrates from multimeric structures—was unknown to impinge directly upon RNP granules. An initial connection was made in 2013 with the recognition that VCP is essential for autophagy-dependent degradation of persistent stress granules, such as those arising from prolonged stress or disease mutations (Buchan et al. 2013). However, the more typical fate of stress granules is disassembly upon removal of stress, whereupon the sequestered mRNPs are returned to the translational pool. Very recently, VCP was found to trigger disassembly of stress granules that arise from short-duration stress (Gwon et al. 2021; Maxwell et al. 2021). In this context, heat shock-dependent polyubiquitination of G3BP1 enables the selective extraction of G3BP1 from stress granules by VCP, causing the stress granule system to fall below the percolation threshold and disassemble. Importantly, disease-causing mutations in VCP result in a failure in stress granule disassembly, providing another example of disease-causing mutations resulting in the accumulation of poorly dynamic RNP granules (Fig. 1E).

### From disturbance of condensate properties to cellular dysfunction

There are two distinct but nonexclusive hypotheses to explain how disease-causing mutations that disturb phase transitions and RNP granule dynamics ultimately lead to cellular dysfunction and demise. First, given the importance of RNP condensates to governing RNA metabolism, and the fact that many of the ALS-FTD-causing mutations in RNA-binding proteins impact mRNPs that are constituents of multiple condensates and traffic between them, one might expect ALS-FTD-causing mutations that alter the dynamics and material properties of RNP granules to cause pleiotropic defects in RNA metabolism. Indeed, evidence of diverse defects in RNA metabolism is prominent in patient-derived tissues and model systems, reflecting abnormalities in multiple aspects of RNA biogenesis, processing, trafficking, utilization, and degradation (Nedelsky and Taylor 2019). A clear example of a link between altered material state and condensate function derives from studies of the impact of *C9ORF72* mutations on the nucleolus. The nucleolus comprises three nested subcompartments that are established through a hierarchy of surface tensions between separate, coexisting phases (Feric et al. 2016). This hierarchical arrangement has been proposed to underlie the vectorial flow of rRNAs and the coordinated modification and sequential assembly of ribosomal subunits (Fatica and Tollervey 2002). This function intersects precisely with the effects of R-polydipeptides arising from expanded *C9ORF72*: as discussed above, R-polydipeptides alter the dynamism of nucleoli, resulting in impaired flow of rRNA from the dense fibrillar component through the granular component and reduced ribosome biogenesis and maturation (Haeusler et al. 2014; Kwon et al. 2014; Lee et al. 2016; White et al. 2019).

Based on this precedent, one might speculate that reduction in the dynamics of condensates, or RNP granule “hardening,” could impair their function and contribute to pleiotropic defects in RNA metabolism in ALS. Indeed, a variety of disease-associated mutations (e.g., in hnRNPA1 [Kim et al. 2013], hnRNPA2B1 [Kim et al. 2013], TIA1 [Mackenzie et al. 2017], TDP-43 [Ding et al. 2021], FUS [Baron et al. 2013], and *C9ORF72* [Lee et al. 2016]) are known to cause hardening of stress granules or RNA transport granules. In those cases where the consequences of this hardening have been investigated, functional defects have been detected. For example, ALS-causing mutations in FUS and TDP-43 promote hardening of RNA transport granules in neurons (Alami et al. 2014; Murakami et al. 2015; Gopal et al. 2017), which correlates with impairment in their microtubule-dependent axonal transport (Alami et al. 2014; Gopal et al. 2017).

ALS-FTD-causing mutations that impair the dynamism of stress granules and RNA transport granules are further linked to impaired local translation, as demonstrated by studies of mutations in FUS (Murakami et al. 2015) and TDP-43 (Nagano and Araki 2021). This effect may occur by disturbing translational buffering, in which condensation serves to control against volatility in the concentration of a key factor—in this case, mRNA (Adekunle and Hubstenberger 2020). In this context, condensates sequester mRNA to control the local availability of mRNA for ribosome binding and translation, and even subtle disturbances in the dynamism of these condensates could result in transcriptomic changes with grave consequences on synaptic activity. Thus, the extent to which condensates harden would be predicted to have the negative consequence of reducing activity, whereas insufficient assembly could lead to hyperactivity. Notably, a role for RNP granule assembly in limiting the availability of mRNAs for local translation is not a new idea, but rather dates back at least 20 years based on circumstantial evidence such as the localization of mRNP granules in synapses, the mRNA contents of these granules, and their behavior in response to synaptic activity (Kosik and Krichevsky 2002). Recent advances in techniques for measuring local translation have substantiated this model and illustrated the inverse relationship between RNP granule assembly and local translation in the distal processes of neurons (Sahoo et al. 2018), lending credence to the hypothesis that impaired dynamism of RNP granules contributes to impaired translation.

Granule hardening may also have loss-of-function consequences. One prominent example is the sequestration of TDP-43 in poorly dynamic RNP granules in the cytoplasm of neurons, which culminates in gradual redistribution of this splicing factor from a predominantly nuclear location to cytoplasmic foci (Honda et al. 2015; Koyama et al. 2016; Fifita et al. 2017). As a result of this mislocalization, such neurons exhibit a depletion of TDP-43 splicing activity in the nucleus, with consequences for the splicing program that lead to neuronal dysfunction. One notable victim of this loss of function is splicing of the transcript encoding stathmin-2, a microtubule-associated protein that is important for axonal structure and function. Depletion of TDP-43 activity leads to retention of a cryptic exon in the stathmin-2 transcript, resulting in the introduction of a neomorphic polyadenylation site, loss of function of stathmin-2 protein, and downstream consequences for axonal function (Klim et al. 2019; Melamed et al. 2019).

The second hypothesis, not mutually exclusive with the first, derives from evidence that the late-stage proteinaceous pathology in ALS-FTD may arise, at least in part, from the evolution of poorly dynamic condensates (Ramaswami et al. 2013). According to this view, poorly dynamic RNP granules promote liquid-to-solid phase transitions in proteins such as TDP-43 that are prone to forming stable or irreversible fibrils. In healthy cells, pathological fibrilization of TDP-43 can be avoided because its steady-state levels are maintained below the threshold for liquid-to-solid phase transition and the assembly of condensates is fleeting. In the setting of disease mutations, however, TDP-43 becomes concentrated in long-lived granules where the risk of pathological phase transition is high. This hypothesis is supported by observations that pathological inclusions exhibit diverse markers of RNP granules and, more importantly, that optogenetic induction of persistent stress granules in cultured neurons leads these granules to evolve to inclusions consistent with late-stage pathology in ALS-FTD patients (Zhang et al. 2019). It is possible that the end-stage pathology of ALS-FTD contributes to the disease process over and above the defects that arise directly from disturbances in condensate function by creating abnormal protein species that acquire neurotoxic properties and perhaps even propagate disease through cell-to-cell spread via a prion-like mechanism.

## CONCLUSION: PROSPECTS FOR MANIPULATING CONDENSATES

Currently, there are no effective therapies for ALS-FTD. Immediate prospects for disease-altering therapeutics in this disease spectrum are largely limited to cases from one of several specific genetic causes where depletion of the offending gene by antisense oligonucleotides offers significant promise. Accordingly, therapies targeting *SOD1* and *C9ORF72* transcripts are currently in development for *SOD1*-related and *C9ORF72*-related diseases, respectively, because substantial depletion of these gene products appears achievable without unacceptable adverse effects (Bennett et al. 2019). Unfortunately, many other genetic targets in ALS-FTD are not accessible in this manner because the gene products serve essential cellular functions. Alternative therapeutic approaches are being pursued, including the identification of modifiers of RAN translation as therapeutic targets to limit the expression of toxic R-polydipeptides arising from *C9ORF72* expansions (Goodman et al. 2019; Yamada et al. 2019; Yuva-Aydemir et al. 2019).

The largest unmet need is found in sporadic ALS-FTD cases that are not linked to a specific genetic defect. Substantial evidence suggests a shared pathomechanism in monogenic and sporadic cases of disease, not the least of which is the common feature in late-stage disease of accumulation of RNA-binding protein inclusions composed predominantly of TDP-43. Additional evidence derives from appreciation that genetic modifiers of certain inherited forms of ALS-FTD (e.g., *ATXN2*) are also found in the sporadic ALS-FTD population. Thus, to the extent that defects in condensation may be an important driver of ALS-FTD in both the monogenic and sporadic populations, it remains possible that reversing an ongoing defect in a condensate could mitigate and perhaps even reverse disease progression.

Considering that condensate properties are a reflection of the topological network of interactions contained within them (Yang et al. 2020), a profound understanding of the underlying network of a given condensate provides a blueprint for how to directly manipulate condensate properties. Indeed, studies of disease-causing mutations have demonstrated the extent to which the assembly of an RNP granule, and its material properties, reflect the sum and strength of all its individual interactions. In the context of disease, a change in one interaction—caused by a point mutation in an RNA-binding protein, the introduction of an LCD-binding peptide, or the expansion of an RNA transcript—can reverberate through the network of interactions, influencing the material properties and function of the condensate. There is reason to be optimistic that this knowledge can be exploited therapeutically. We now have increasingly refined catalogs of the constituents present in many condensates. In at least one disease-relevant case, the key network of interactions that define the percolation threshold and control condensate material properties has been defined (Yang et al. 2020). Indeed, genetic manipulation of this network by inhibiting key proteins in this network causes predictable consequences on condensate assembly and dynamics (Yang et al. 2020). Thus, in disease situations, it should be possible to design therapies (e.g., small molecules or antisense oligonucleotides) that target these percolation networks to restore homeostasis.

Insights from the study of VCP provide an additional approach to modulating condensate properties, where targeting those factors that exert higher-order regulation of condensation (e.g., by post-translational modification of condensate constituents) can provide an entry point for therapeutic manipulation (Gwon et al. 2021; Maxwell et al. 2021). Indeed, targeting the kinase responsible for phosphorylation of VCP accelerates stress granule disassembly (Wang et al. 2019).

Beyond ALS-FTD, a role for pathological phase transitions is increasingly implicated in other neurodegenerative diseases. The idea that defects in biomolecular condensation may contribute to neurodegeneration more broadly arises from the observation that other important disease-associated proteins, namely tau (Ambadipudi et al. 2017; Zhang et al. 2017; Wegmann et al. 2018), α-synuclein (Ray et al. 2020), and the prion protein PrP^C^ (Kostylev et al. 2018), are also capable of LLPS in addition to their well-known liquid-to-solid phase transitions. Indeed, in some instances disease-causing mutations have been documented to alter phase separation behavior of these proteins, suggesting that pathological phase transitions may also contribute to initiating diseases such as Alzheimer's disease, Parkinson's disease, FTD-TAU, and prion disease. Further research will be needed to determine the normal role of phase transitions in these proteins, if any, and the extent to which corruption of these phase transitions contributes to cellular dysfunction.
